# Screening, Identification, and Quantification of Nutritional Components and Phytochemicals in Foodstuffs

**DOI:** 10.3390/foods10010125

**Published:** 2021-01-08

**Authors:** Dario Donno

**Affiliations:** Dipartimento di Scienze Agrarie, Forestali e Alimentari, Università degli Studi di Torino, 10095 Grugliasco (TO), Italy; dario.donno@unito.it; Tel.: +39-011-670-8751

Foods confer many health-promoting benefits to humans for the treatment/prevention of different diseases. The interest in bioactive compounds, nutritional components, and phytochemicals in potential health-promoting foods continues to grow, powered by the identification of health-promoting properties and potential applications of nutraceutical substances. These products may range from isolated nutrients, agri-foods, dietary supplements, and diets to genetically engineered “designer” foods, herbal products, and processed foods. Today, nutraceutical products may be both traditional foods (e.g., meat products, fish, vegetables, fruits, chocolate, grains, and tea) and non-traditional foods (e.g., added ingredients or products derived from agricultural breeding). These products have become very attractive to the food industry, prompting their use as replacements for synthetic chemicals and nutraceuticals, but natural resources are suffering from less attention and research, and their nutritional, economic, and socio-cultural potentials are not fully exploited.

Studies on natural resources and foodstuffs are very important to discover new sources for natural antioxidants, health-promoting materials, nutraceuticals, and potential functional foods. The identification and quantification of nutritional substances and bioactive compounds in foods and the evaluation of their biological activities are important to gauge their efficacy as dietary interventions and in healthy applications. In the last few years, studies in the field of natural biomolecules and health-promoting foods have been focusing on naturally health-positive molecules available in food, herbal preparations, plants, and derived products. Their utilization in foodstuffs could increase quality, safety, and added value; for this reason, traditional and innovative techniques for extraction/purification and identification/quantification of bioactive compounds and nutritional substances using eco-friendly analytical strategies need to be stimulated and developed to improve production yields.

This Special Issue provides readers with a good overview of the status and important insights and developments in this field. It includes review and research papers focused on modern analytical approaches, traditional and innovative techniques, and biological tests applied to the screening, identification, and quantification of nutritional components and phytochemicals in foodstuffs together with the evaluation and valorization of innovative natural sources of bioactive molecules and relative health-promoting properties.

Turrini et al. [1] investigated, for the first time, an alternative method to produce *Ribes nigrum* bud derivatives, a category of botanicals marketed as plant food supplements in the European community. Pulsed ultrasound-assisted extraction (PUAE), using a food-grade solvent according to green chemistry principles, was employed and compared to the conventional extraction method. Untargeted polyphenolic fingerprints (UV-Visible and fluorescence) coupled with chemometrics were employed to quickly screen the best extraction conditions, evaluated by the design of experiment (DoE) method. The polyphenolic fraction of the optimized PUAE extract was quantified by targeted HPLC fingerprint and its antiradical activity was determined. PUAE on a lab pilot reactor was proven to be the most practical approach for a rapid (20 min vs. 21 days maceration) and efficient extraction of bioactive polyphenolics from *Ribes nigrum* buds, encouraging the scale up to an industrial scenario.

Jang et al. [2] studied the optimization and validation of an analytical method for separating flavonoid isomers in common buckwheat sprout extract (CSE). Factors such as range, linearity, precision, accuracy, the limit of detection, and limit of quantification were evaluated for each standard using high-performance liquid chromatography (HPLC). Based on resolution and symmetry, a column temperature of 40 °C with 0.1% (*v*/*v*) acidic water and acetonitrile as mobile phases, at a flow rate of 1 mL/min were determined to be the optimal analytical conditions. The developed method was used to analyze flavonoids in CSE, with isomers satisfactorily separated and simultaneously quantified.

Lavefve et al. [3] determined the stability of polyphenolics in five products (ice pop, oatmeal bar, graham cracker cookie, juice, and gummy product) prepared with wild blueberry (WBB) powder that can be added to the formulation of foods to encourage consumption of health-promoting polyphenolics. Samples stored at 21 °C, 4.4 °C, or −20 °C (ice pops only) were analyzed at 0, 2, 4, 6, and 8 weeks for polyphenolic content and percent polymeric color. Total anthocyanins decreased over storage and storage temperatures in all products. However, the ice pop and the refrigerated juice both retained over 90% of their initial total anthocyanin content. The refrigerated oatmeal bar also showed good retention of anthocyanins (86%), but the gummy product retained only 43% and 51% when stored at 4.4 °C or 21 °C, respectively. The lower amount of polyphenolic compounds recovered in the gummies stored at 4.4 °C compared to 21 °C may be attributed to reduced extraction efficiency as a result of gel hardening at refrigerated temperature. Chlorogenic acid and flavonols were generally more stable than anthocyanins throughout storage.

Dini et al. [4] validated, for the first time, a highly specific automated enzymatic method to quantify the acetic acid in wine vinegar, in terms of linearity, precision, repeatability, and uncertainty measurement. The results were compared to the Community method of analysis. Regression coefficient ≅ 1 and the normal distribution of residuals in the ANOVA test confirmed the method’s linearity. Lower limit of detection—LLOD (0.946 ppm) and lower limit of quantification—LLOQ (2.00 ppm) defined the method’s sensitivity. The results of the tested and the Community methods, linearly distributed in the Shapiro–Wilk test, confirmed the method’s repeatability. The few anomalous data in the Huber test were due to random errors. The high selectivity of the enzymatic method, which exclusively measures acetic acid concentration, determined the significant differences between the two tests, examined in the accuracy determination. The enzymatic method can be considered applicable since its precision and uncertainty were lower than the Community method values (relative percentage deviations = 10%). The enzymatic method compared to the Community method reduces the analysis time and the risk of errors due to operators (avoiding pipetting errors and wrong calculations), minimizes solvent and the sample consumption, and guarantees assay quality through method standardization.

Bonasia et al. [5] assessed the chemical composition (minerals, organic acids, free sugars, volatile, and phenolic compounds) of six garlic landraces collected from Puglia Region (Foggia Province) along with their main morpho-biometrical traits. A commercial genotype was also considered as a reference standard. The landraces showed large variability, but in general high morphological standards, high levels of cations and phenols, and low levels of volatile-(S)-compounds in comparison with the commercial genotype and the literature values. “Aglio di Peschici” and “Aglio Rosso di Monteleone di Puglia” were very rich in minerals and phenols (mainly ferulic acid and iso-rhamnetin). The increase in knowledge of the chemical properties of these garlic landraces could represent a tool for encouraging the consumption of a food product. At the same time, the consumption of these landraces would stimulate their cultivation and could highly contribute to protection against the risk of erosion of agro-biodiversity through *in situ*/on-farm conservation.

Beccaro et al. [6] investigated chestnut cultivars grown in the same pedoclimatic conditions and on the same clonal rootstock. Chestnuts were characterized by sensory, spectrophotometric, and chromatographic analysis to determine the phytochemical composition and nutraceutical properties. A multivariate approach, including principal component analysis and conditional inference tree models, was also performed. The multivariate approach showed that phenolic acids and tannins were the bioactive classes with the highest discriminating power among different genotypes, and that genotype is a significant variable (*p* < 0.05). Furthermore, most of the analyzed chestnut cultivars showed a content of bioactive compounds similar to or higher than the main hazelnut, walnut, and almond varieties. Chestnut agrobiodiversity could be intended as strictly associated to the genotype effect and underlines the large variability within the genus *Castanea*, and therefore, the importance of in farm and ex situ conservation of local germplasm is part of a global strategy aimed at increasing the levels of agrobiodiversity.

Suleria et al. [7] determined the polyphenol content and their antioxidant potential in twenty different fruit peel samples in an ethanolic extraction, including their comprehensive characterization and quantification using the LC-MS/MS and HPLC. The application of LC-ESI-QTOF-MS/MS tentatively identified and characterized a total of 176 phenolics, including phenolic acids (49), flavonoids (86), lignans (11), stilbene (5), and other polyphenols (25) in all twenty peel samples. From HPLC-PDA quantification, the mango peel sample showed significantly higher phenolic content, particularly for phenolic acids and flavonoids, as compared to other fruit peel samples. These results highlighted the importance of fruit peels as a potential source of polyphenols. This study provided supportive information for the utilization of different phenolic rich fruit peels as ingredients in food, feed, and nutraceutical products.

Krawęcka et al. [8] assessed the effect of adding 0%, 5%, 10%, 15%, and 20% oat (1,3)(1,4)-β-D-glucans to physicochemical properties, as well as the cooking and sensory qualities of durum wheat pasta. Additionally, 5% of xanthan gum and vital gluten was added to improve the cooking and sensory qualities of pasta. The study showed that the addition of β-glucans led to an increase of the water absorption index (WAI), water solubility index (WSI), and viscosity of products. At the same time, an increase in the content of fat, ash, and dietary fiber was observed. The addition of (1,3)(1,4)-β-D-glucans influenced the cooking quality of the pasta, extending the minimum cooking time and increasing the loss of dry matter. At the same time, the color of the product changed. In the case of cooked pasta, the addition of β-glucans decreased the brightness and increased the yellowness and redness. It was found that the products enriched with 10–15% of β-glucans, as well as 5% of xanthan gum and vital gluten would yield functional pasta that may offer health benefits beyond its nutritional value. Furthermore, this could influence high cooking and sensory quality.

Müller et al. [9] evaluated the micro- and macronutrient composition of representative, randomly mixed samples of the 15 different hazelnut cultivars. Protein, fat, and fiber contents were determined using established methods. Fatty acids, tocopherols, minerals, trace elements, and ultra-trace elements were analyzed using gas chromatography, high-performance liquid chromatography, and inductively coupled plasma triple quadrupole mass-spectrometry, respectively. The different hazelnut varieties contained valuable amounts of fat, protein, dietary fiber, minerals, trace elements, and α-tocopherol, however, in different quantities. The variations in nutrient composition were independent of growth conditions, which were identical for all hazelnut varieties. Therefore, each hazelnut cultivar presented a specific nutrient profile.

Distefano et al. [10] addressed the effects of two storage temperatures, namely 10 °C (T10) and 20 °C (T20), on main quality and functional traits of three cherry tomato cultivars (“Eletta”, “Sugarland”, and “Ottymo”), after 0 (S0), 7 (S7), and 14 (S14) days of storage. At T10 both fruit weight and firmness were better retained during storage. At S14, T10 promoted fruit Chroma and overall fruit color deviation (ΔE*ab). Total polyphenols content (TPC) of fruits peaked at S7 then declined at S14 (by 16%), with the highest values recorded at T10. Lycopene showed a similar trend, but with a higher average concentration recorded at T20. β-carotene content peaked at S14, irrespective of the storage temperature. At S14, the concentrations of phytoene and phytofluene were higher at T20, but the opposite was found at S7. “Sugarland” and “Ottymo” showed the highest ΔE*ab during storage, with the former cultivar proving to have the highest TPC and lycopene content, whereas “Eletta” did so for phytoene and phytofluene. The results suggested that unravelling the possible functional interactions among these three carotenoids would allow for a better orientation of breeding programs, targeting the phytochemical evolution of tomatoes during refrigerated storage.

Donno et al. [11] investigated the main analytical strategies commonly utilized in the agri-food industry, often using complementary technologies with different purposes. This overview presented the most recent MS-based techniques applied to food analysis. An entire section was dedicated to the recent applications of high-resolution MS. Covered topics included liquid (LC)– and gas chromatography (GC)–MS analysis of natural bioactive substances, including carbohydrates, flavonoids and related compounds, lipids, phenolic compounds, vitamins, and other different molecules in foodstuffs from the perspectives of food composition, food authenticity, and food adulteration. The results represented an important contribution to the utilization of GC–MS and LC–MS in the field of natural bioactive compound identification and quantification.

In recent years, the agri-food industry has applied new analytical strategies for a full characterization of foods since consumers and regulatory agencies required a molecular characterization. Validated analytical approaches using high-performance systems were improved to ensure traceability and quality of food. Chromatographic techniques coupled to suitable detection strategies are one of the most effective tools to separate the single molecules and develop a specific profile (“fingerprint”). If chromatography, coupled to detection systems as mass spectrometry, is further combined with chemometrics, clearer patterns might be developed for analytical fingerprints.
